# Cryotherapy in extra-abdominal desmoid tumors: A systematic review and meta-analysis

**DOI:** 10.1371/journal.pone.0261657

**Published:** 2021-12-23

**Authors:** Bimal Mayur Kumar Vora, Peter L. Munk, Nagavalli Somasundaram, Hugue A. Ouellette, Paul I. Mallinson, Adnan Sheikh, Hanis Abdul Kadir, Tien Jin Tan, Yet Yen Yan

**Affiliations:** 1 Department of Diagnostic Radiology, Singapore General Hospital, Singapore, Singapore; 2 Musculoskeletal Section, Department of Radiology, Vancouver General Hospital, University of British Columbia, Vancouver, BC, Canada; 3 Division of Medical Oncology, National Cancer Centre Singapore, Singapore, Singapore; 4 Health Services Research Unit, Singapore General Hospital, Singapore, Singapore; 5 Department of Radiology, Changi General Hospital, Singapore, Singapore; University of Texas M. D. Anderson Cancer Center, UNITED STATES

## Abstract

**Introduction:**

Desmoid tumor is a locally-invasive neoplasm that causes significant morbidity. There is recent interest in cryotherapy for treatment of extra-abdominal desmoid tumors. This systematic review assesses evidence on safety and efficacy of cryotherapy in the treatment of extra-abdominal desmoid tumors.

**Materials and methods:**

The systematic review was conducted with reference to the Preferred Reporting Items for Systematic Reviews and Meta-Analyses (PRISMA) statement. Literature search was performed using MEDLINE and the Cochrane Central Register of Controlled Trials. 9 full text papers were reviewed and meta-analysis was performed for measures of safety, efficacy and symptom relief.

**Results:**

The estimated pooled proportion of major and minor complications was 4.2% (95% CI, 1.8–9.6; I ^2^ = 0%) and 10.2% (95% CI, 5.7–17.8; I ^2^ = 0%) respectively. The estimated pooled proportion of non-progressive disease rate of all studies was 85.8% (95% CI, 73.4–93.0; I ^2^ = 32.9%). The estimated progression free survival rate at 1 year was 84.5% (95% CI:74.6–95.8) and 78.0% at 3 years (95% CI: 63.8–95.3). As for pain control, the estimated pooled proportion of patients with decrease in visual analogue scale (VAS) > = 3 for those with VAS > = 3 before treatment for 2 studies was 87.5% (95% CI, 0.06–100; I ^2^ = 71.5%) while 37.5% to 96.9% of patients were reported to have experienced partial or complete symptom relief in the other studies.

**Conclusion:**

Cryotherapy is a safe and effective treatment modality for extra-abdominal desmoid tumors with efficacy similar to those treated with traditional strategies in the short to medium term.

## Background

Desmoid tumor (DT) is a monoclonal fibroblastic proliferation that typically occurs in the 3^rd^ and 4^th^ decade of life. The locally-invasive nature of these tumors can cause considerable morbidity. Recurrence rate is high (>60%) after surgical resection and spontaneous regression is documented in 20% of patients [1, 2]. DT mostly occur sporadically, with β-catenin gene mutations found in approximately 85%–90% of tumors [3]. 5–15% of DT can arise in patients with familial adenomatous polyposis (FAP) syndrome, characterized by an APC gene mutation [4].

The National Comprehensive Cancer Network guidelines recommend initial active surveillance in the absence of progressive, morbid, or symptomatic disease and intervention in DT showing ongoing progression with potential morbidity and significant symptoms [5–8]. Although radiotherapy effectively controls desmoid tumors in most cases, there are concerns of late toxicity [9]. Despite the disputed evidence, most guidelines suggest the use of tamoxifen with or without non-steroidal anti-inflammatory drug (NSAIDs) as first line medical treatment [10, 11]. Response rates of chemotherapy are between 35 to 40%, and usually occur months after commencement of therapy [12]. Sorafenib has demonstrated significant benefit when compared to placebo, with a median time to response of 9.6 months [2]. The results of ongoing phase III trials with Nirogecestat, an agent targeting the Notch signaling pathway, are pending [13]. Hence, there remains a need for treatment options that would allow rapid control of symptoms in patients with DT.

Image-guided interventional radiologic techniques such as alcohol injection and electroporation [14], high-intensity focused ultrasound [15, 16], and percutaneous microwave and radiofrequency ablation have been used in the treatment of DT [17–19]. Cryoablation has recently been shown in studies to be efficacious and safe [20–28].

With local control techniques gaining traction as a treatment option for tumor debulking or cure, the role of cryotherapy needs to be assessed further. The aim of this study is to evaluate current evidence on the efficacy and safety of cryoablation for treatment of DT.

## Material and methods

### Search strategy

A systematic review was conducted with reference to the Preferred Reporting Items for Systematic Reviews and Meta-Analyses (PRISMA) statement [29]. Two reviewers independently performed a literature search by using MEDLINE and the Cochrane Central Register of Controlled Trials to identify relevant articles till 14^th^ June 2021. Search terms used were “desmoid tumor”, “aggressive fibromatosis”, “percutaneous cryoablation” and “percutaneous cryotherapy”. Abstracts were screened for relevance, and full-text articles were retrieved. These were independently analyzed and assessed for eligibility by both reviewers. The reference lists of these articles were studied to identify additional relevant papers. All conflicts were resolved in consensus.

### Inclusion criteria

Studies matching the following selection were included:

- Cohort ≥ 10 patients;
- Studies performed with curative intent (complete ablation) and/or palliative intent (partial ablation) as defined according to the commonly agreed standardized terminology and reporting criteria [30];
- Published in English;
- Animal studies were excluded;
- No date limitations and no other limitations on study type;
- No restriction on the type of cryoprobe design used in the studies;

### Data extraction

Data extraction was performed independently by two reviewers using a predefined data extraction template with data fields including complications, symptom relief, and survival.

### Quality assessment

The Downs and Black checklist, an assessment tool for quality of randomized and nonrandomized studies with 27 checklist items, was modified to allow it to be used to assess study quality for the present series of nonrandomized studies [31], similar to modifications used by other studies [32]. (Appendix A in S1 File) With this modified score, a maximum score of 14 was possible, with higher scores reflecting higher quality of studies and lower risk of bias, and lower scores indicating higher risk of bias. Evaluating the risk of bias among the studies was performed to assess anticipated variability in either the results or validity of the included studies.

### Statistical analysis

Reported results were pooled using random-effects model with adjustments of confidence interval from the Knapp-Hartung method. Statistical heterogeneity was assessed using the *I*^2^ statistic. For pre-ablation tumor volume and diameter, generic inverse variance method was used. Estimation of variation in 2 studies [23, 25] was performed based on Wan et al. [33]. For complications and non-progressive disease rates, logit-transform proportions with generalized linear mixed-effects model were applied. For progression-free survival, coordinates of the published Kaplan-Meier curves were extracted using WebPlotDigitizer 4.4 [34]. Individual participant data was reconstructed using an algorithm by Guyot et al. [35], and combined to produce one survival curve.

For risk of bias assessment, funnel plots were generated for the meta-analyses that have at least 6 studies (Appendix B in S1 File). All analyses were conducted using R 4.0.2 and the *meta* package.

## Results

**Fig 1:**
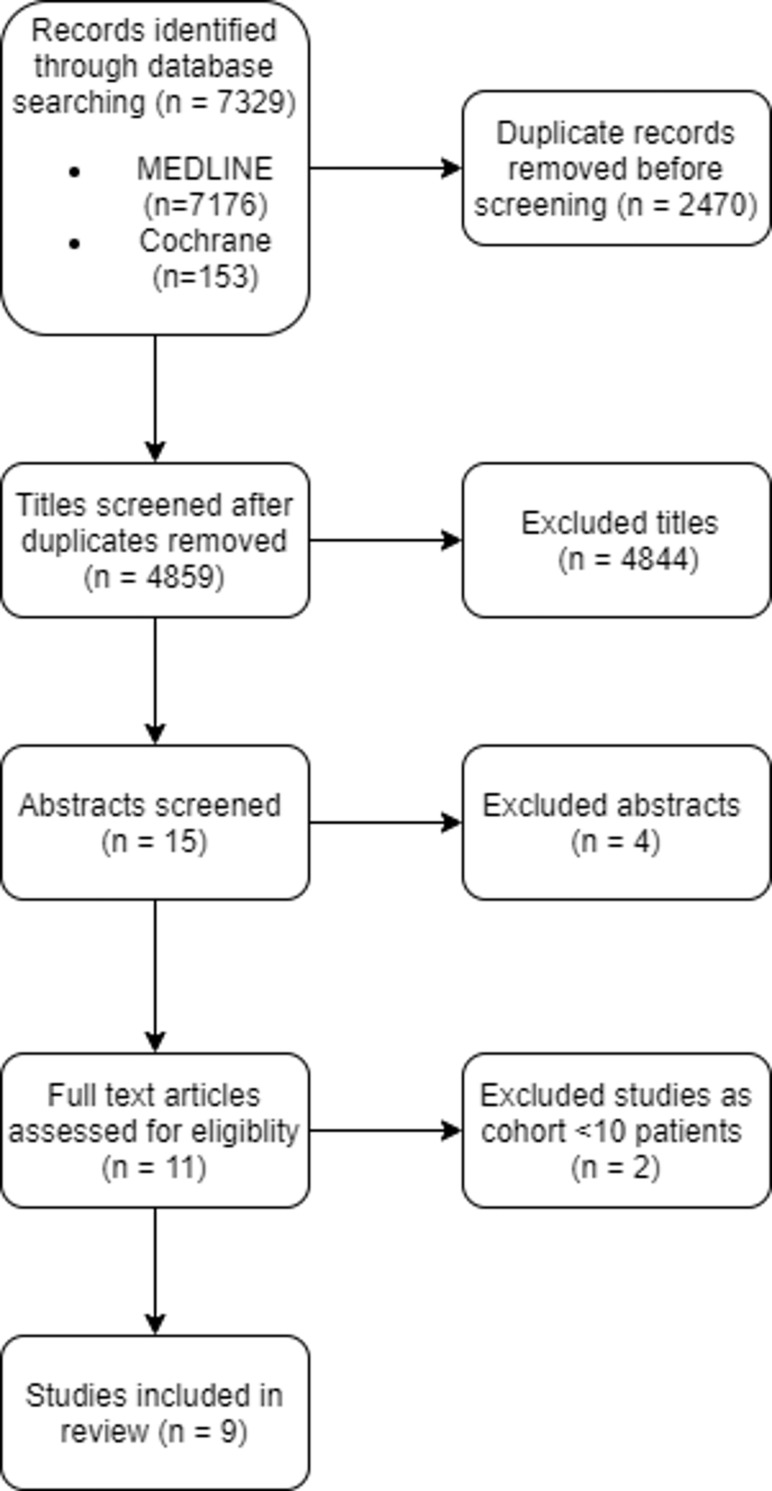
Search strategy according to PRISMA template.

The literature search retrieved a total of 7329 titles (7176 titles from MEDLINE and 153 titles from Cochrane Central Register of Controlled Trials), 2470 duplicates, (4859 titles without duplicates), of which 15 abstracts were screened. (Fig 1) 9 full text papers (non-randomized studies) were reviewed and all met the inclusion criteria. Apart from a prospective phase 2 study, the rest were retrospective studies. There was no overlapping patient cohort from the same center. No study scored higher than 12 of 14 on quality assessment per the modified Downs and Black checklist and the mean score was 10.4. Inclusion and exclusion criteria of each study are summarized in Table 1. Asymmetrical patterns in all the funnel plots, most pronounced in the pre-ablation tumor volume, might be indicative of publication bias (Appendix B in S1 File).

**Table 1:** Inclusion and exclusion criteria reported by each included paper.

Study, Country of Origin, Year	Inclusion criteria	Exclusion criteria
Yan, Canada, 2021	•Biopsy proven progressive or symptomatic extra-abdominal DT and at least one cryoablation treatment between 25 February 2010 and 25 February 2020, with data available for follow up through February 2020.	N.R.
	• Pediatric patients defined as less than 18 years old.	
	• Patients with more than one treated tumor or who were treated in the same location more than once were included as a separate encounter	
Efrima, Israel, 2021	• Patient over 18 years of age that had been treated with percutaneous cryosurgery by means of a three-phase protocol.	N.R.
	• Extra-abdominal desmoid tumors that showed progression of size in at least two sequential MRI scans executed 3 months apart.	
	• Patients with symptomatic desmoid tumors.	
Auloge, France, 2021	• All patients with extra abdominal DT who underwent cryoablation from Jan 2008 to Nov 2019 were identified by research performed in our institutional Radiological Information System (Xplore, EDL, la Seyne-sur-Mer, France) with “Cryoablation” and “Desmoid tumor” entered together.	• Patients involved in a prospective study
Kurtz, France, 2021	• Pathologically confirmed extra-abdominal DT.	• Contraindication for the procedure as stated by the interventional radiologist in terms of tumor size, proximity to neural/vascular structures making the procedure involving unacceptable risk
	• Progressive disease after at least two lines of adequate medical therapy [including tamoxifen, NSAIDs or chemotherapy], with functional symptoms and/or pain.	
	• Unresectable tumor or tumor amenable only to mutilating surgery deemed inappropriate in a NETSARC tumor board.	
	• Patients with mRECIST 1.1 criteria stable disease, but with persistent functional disability or tumor-induced pain not controlled by adequate pain medication including narcotics patients.	
	• Other criteria included: age ≥ 18 years old; tumor deemed accessible for cryoablation procedure by the radiologist operator of the investigating center (with 90% of destruction of the tumor achievable in one procedure of cryoablation and with a possible second cryoablation procedure if necessary and if scheduled at study entry); measurable lesion (mRECIST 1.1) using MRI (gadolinium injection mandatory); ECOG performance status 0–2; adequate biological and hematological parameters; affiliation to a medical insurance scheme for health costs coverage, and signed written informed consent.	
		• Impaired hemostasis
		• Concomitant participation in other experimental studies that could affect end-points of this study
		• Contraindication to any form of sedation, MRI or gadolinium injection [proven allergy, glomerular filtration rate <30 ml/min by Modification of Diet in Renal Disease formula]
		• Psychiatric disorders and adults under guardianship, pregnancy or breastfeeding, or under judicial protection.
Saltiel, Switzerland, 2020	• Patients with histologically confirmed extra-abdominal DT treated with cryoablation and followed up with MRI before and after treatment.	• Intra-abdominal DT or no available follow-up.
Bouhamama, France, 2020	• Extra-abdominal DT that was histologically proven by a percutaneous or a surgical biopsy.	N.R.
	• Surgery was contraindicated because it was considered too mutilating.	
	• Tumors were clinically or radiologically progressive despite systemic treatment (tamoxifen, pazopanib or non-steroidal anti-inflammatory drugs).	
	• Tumors were considered by a senior interventional radiologist as accessible to percutaneous cryoablation on the preoperative imaging: visible with ultrasound or CT scan during procedure.	
	• Patients were included regardless of the size of the tumor.	
Tremblay, USA, 2019	• Biopsy proven extra-abdominal DT who were treated with percutaneous cryoablation from July 2014 to May 2018, with follow up through January 2019.	N.R.
Schmitz, USA, 2016	• Patients with extra-abdominal DT who underwent percutaneous cryoablation between June 15, 2004, and June 15, 2014.	N.R.
Havez, France, 2013	• Presence of a symptomatic histologically-proven extra-abdominal DT	N.R.
	• Percutaneous cryoablation technically possible and en-bloc resection not possible (due to lesion location or size) or refused by the patient	
	• Cryoablation treatment option approved in a multidisciplinary team meeting.	

Abbreviations: DT: desmoid tumor: NSAIDs: non-steroidal anti-inflammatory drugs; ECOG: Eastern Cooperative Oncology Group; N.R.: not reported.

### Data collection

The following data were extracted: study design; cohort size; number of patients; mean age; average follow-up; mean lesion size; complete or partial ablation, technical success; response by radiological criteria, complications and symptom improvement.

### Demographics

A total of 214 patients with 234 extra-abdominal DT underwent 282 cryoablation procedures from 2004 to 2020, with at least 2 procedures performed for 30 tumors. (Table 2) Upper and lower limbs were the most common sites (80 tumors, 34.1%), followed by chest wall (50 tumors, 21.3%) and pelvic girdle (36 tumors, 15.3%). Other sites include the shoulder girdle (26 tumors, 11.1%), paraspinal region (25 tumors, 10.6%) and abdominal wall (17 tumors, 7.2%).

**Table 2:** Demographics and outcomes following cryoablation for desmoid tumors.

Study, Country of Origin, Year	No. of Patients/Tumors/Procedures	Mean age (year)	Sex (M/F)	Curative/ Palliative Intent (patients)	Median Follow-up	Technical Success (%)	Major Complication (%)	Median Change in Tumor Volume	Progression Free Survival	Non-progressive disease rate	Symptom relief
Yan, Canada, 2021	25/26/44	32	8/17	10/15	Imaging and clinical follow up is 6M and 10M respectively; Follow up for DFS tumor recurrence and symptom recurrence is 15.3 and 21.0M respectively.	100	2.4	4-6M: TLV: -6.8%(NS) VTV: -44.2% (p<0.05) 7-12M: TLV: -6.7% (NS); VTV: -43.7% (p<0.05)	3Y: 82.9% (tumor progression); 78.4% (symptom recurrence); Median DFS for tumor progression and symptom recurrence were not reached.	10-12M: 92.3%	96.9%
Efrima, Israel, 2021	11/11/16	35.3	5/6	N.R.	6M	N.R.	0	At last follow up: TLV: -27.9% VTV: -64.7%	N.R.	N.R.	81%
Auloge, France, 2021	30/30/34	39^b^	9/21	19/11	18.5M	N.R.	13.3	At last follow up: VTV: − 80% 1Y: TLV: -66.6% 3Y: TLV: −76.4%	1Y: 85.1% 3Y: 77.3%	At last follow up: 83.3%	96.7%
Kurtz, France, 2021	50/50/55	41	11/39	N.R.; all patients aimed to have at least 90% tumor destruction	31M (DFS)	N.R.	15 of 50 patients had grade 3 or 4 side effects. No grade 5 side effects	N.R.	Median DFS not reached at median follow up	12M: 86%	Significantly improved functional status and pain scores (BPI and EQ5D-3L)
Saltiel, Switzerland, 2020	10/10/14	33	1/9	8/2	53.7M^a^	N.R.	14.2	6M: Curative intent: TLV: -16.3% VTV: -44.1%^a^ Palliative intent: TLV: +113.2% VTV: +148.6%^a^ 12M: Curative intent TLV: -28.8% VTV: +103.4%^a^ Palliative intent: TLV: +112.9% VTV: +192.1%^a^	3M: 90% 6M & 12M: 62%	12M: 54.5%	37.5%
Bouhamama, France, 2020	34/41/41	38	9/25	12/22	25M^a^	N.R.	4.8	6M: TLV: -12.4% VTV: -65.6% (p<0.05)^a^	3Y: 42.2%	6M: 73.5%	Decreased VAS score at 6 months
Tremblay, USA, 2019	23/23/30	40.5^b^	9/14	12/11	16.8M^a^	100	6.7	12M: TLV: -56% VTV: -80% ^a^	N.R.	12M: 100%	90%
Schmitz, USA, 2016	18/26/31	39.9	8/10	18/0	16.2M^a^	100	0	At last follow up: TLV: -89.2%	N.R.	At last follow up: 95.7%	80%
Havez, France, 2013	13/17/17	39.3	4/9	9/8^c^	7M	100	5.8	3M: TLV: -56%, 6M: TLV: -73.5%, At last follow up: TLV: -87%	6M, 12M & 24M: 82.3%	At last follow up: 88.2%	76.9%

Abbreviations: M: months; Y: years; NS: not significant; N.R.: not reported; DFS: disease free survival; BPI: Brief Pain Inventory; TLV: total lesion volume; defined by all the authors as volume of the tumor measured by ellipsoid formula, manual segmentation or software assisted, VTV: viable tumor volume; defined by all the authors as volume of the enhancing portions of the tumor measured by ellipsoid formula, manual segmentation or software assisted.

^a^ Mean value

^b^ Median value

^c^ as per procedure.

15 patients in the cohort had FAP. The number of tumors per patient was reported in all studies, with a mean of 1.1 tumors per patient.

7 studies recorded both the pre-ablation mean tumor diameter and volume [20, 22–25, 27, 28], while 1 study recorded only the pre-ablation tumor volume [26] and 1 study only recorded pre-ablation tumor diameter [21]. Volume was assessed in different ways, either by an ellipsoid formula, a modified ellipsoid formula (½ x (length x width x height)), manual segmentation or automated software measurement. 2 studies utilised the ½ x (length x width x height) formula [20, 27], 1 study utilised the ellipsoid formula [22], 3 studies utilised manual segmentation [23, 25, 28] and 1 study used IntelliSpace Discovery software platform (Philips Healthcare) semi-automatic 3D segmentation tool [26]. 1 study did not mention the method of volume measurement [24].

**Fig 2:**
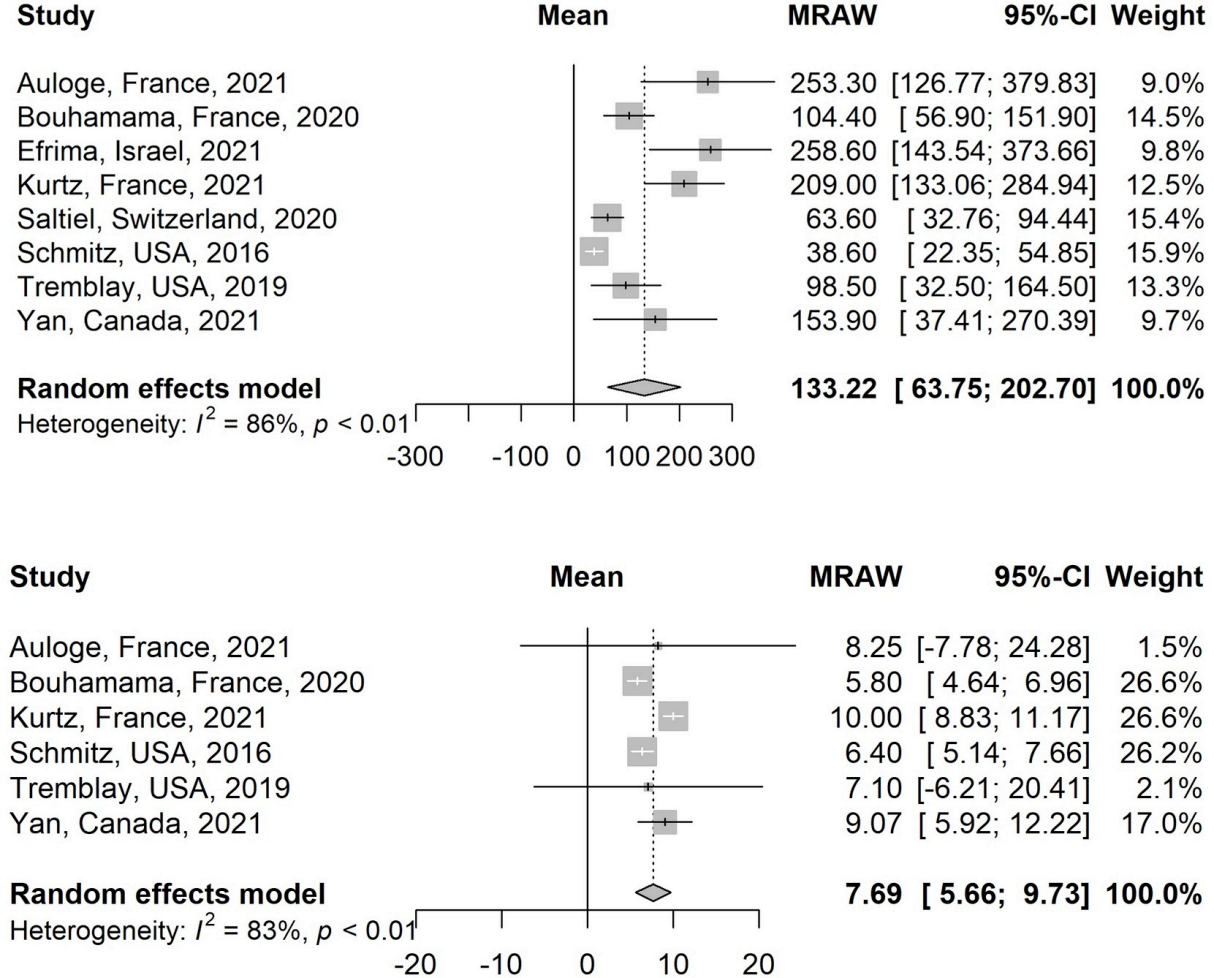
Comparison of the pre-ablation tumor volume (Fig 2A) and diameter (Fig 2B) across the selected studies.

The estimated pooled mean of pre-ablation tumor volume in the selected 8 studies is 133.22 cm3 (95% CI, 63.75–202.70; I^2^ = 85.7%) (Fig 2A) [20, 22–28].

The estimated pooled mean of pre-ablation tumor diameter in the selected 6 studies is 7.69 cm (95% CI, 5.66–9.73; I^2^ = 83.1%) (Fig 2B).

### Indications for cryoablation and patient selection

Patient selection criteria for cryoablation was heterogeneous among studies with palliative or curative intent. From the applicable studies, 79 and 61 patients were treated for curative and palliative intent respectively [20, 22, 23, 25, 27, 28]. 38 patients underwent cryoablation as 1st line therapy and 191 patients underwent cryoablation as >1st line therapy.

There was variability in patient selection criteria for cryoablation in the included studies, with some studies only including progressive DT [23, 24, 27], whereas others included DT undergoing cryoablation as first line and >1first line treatment [20–22, 25, 26, 28].

In this cohort, previous treatment received includes: previous surgery (n = 63), NSAIDs (n = 72), chemotherapy (n = 35), radiotherapy (n = 13), selective estrogen receptor modulator (SERM) (n = 33) and tyrosine kinase inhibitor (n = 16).

### Cryoablation technique

All patients underwent preoperative imaging, namely CT and MRI. Amongst all studies, 3 patients had ultrasound-guided open surgery cryoablation [25], while percutaneous cryoablation was performed for the rest of the patients. One patient underwent pre-cryoablation tumor embolization of a hypervascular tumor [27]. General anesthesia (n = 255), conscious sedation (n = 22) and local anaesthesia (n = 3) were administered for cryoablation. Percutaneous cryoablation procedures were CT or MRI guided with or without ultrasound supplementation. Efrima et al. utilized a 3-phase protocol consisting of ablation software with 3-dimensional model assistance that aids in pre-operative planning, navigated cryoprobe placement and volumetric tissue measurement and characterization [26].

Mean number of cryoprobes used ranged from 2.4 [21] to 9.6 [26]. All cryoablation protocols consists of 2 consecutive freeze-thaw cycles with minor variations in freeze and thaw type (active and/or passive) and times. Freezing times were mostly 10 minutes and thaw times were mostly between 5–10 minutes.

Technical success was 100% in 4 articles [20–22, 28] and not reported in 5 articles [23–27].

### Safety

**Fig 3:**
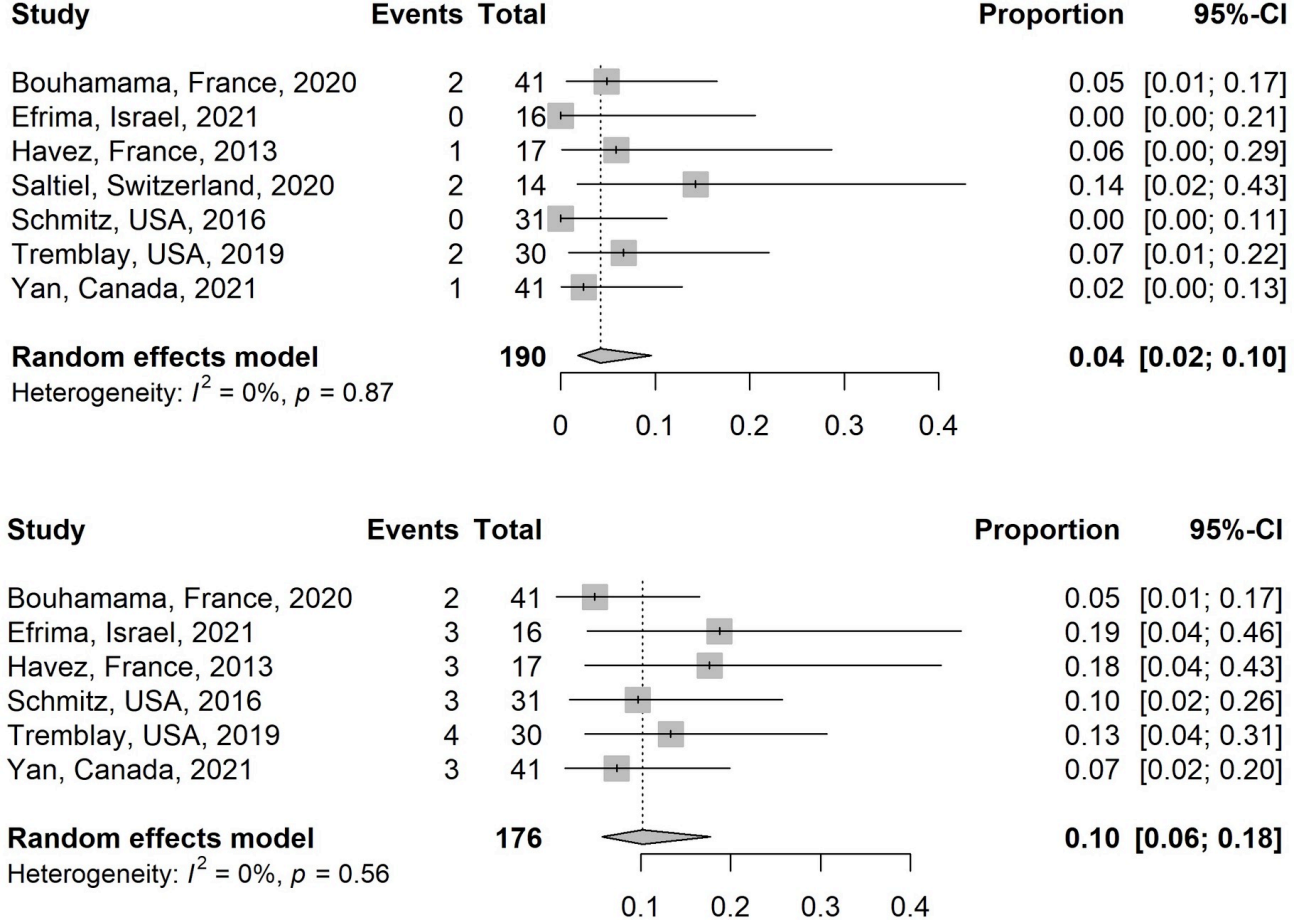
Comparison of the major (Fig 3A) and minor (Fig 3B) complications across the selected studies.

The literature generally classified complications into major and minor complications and used varied scoring systems. There were no deaths amongst this series. Major and minor complications ranged from 2.4% to 14.2% and 4.8% to 23.3% respectively for all procedures. The estimated pooled proportion of major and minor complications in the selected studies is 4.2% (95% CI, 1.8–9.6; I^2^ = 0%) (Fig 3A) and 10.2% (95% CI, 5.7–17.8; I^2^ = 0%) respectively (Fig 3B) [21–23, 25, 27, 28].

The most common major complication reported was nerve injury (n = 10), which included common peroneal nerve injury (n = 5), sciatic nerve injury (n = 1), brachial plexopathy (n = 1), paresthesia/dysthesia/neural impairment (n = 3). Some of the other major complications included rhabdomyolysis (n = 7), skin necrosis (n = 3), bleeding (n = 2), infection (n = 1) and colo-cutaneous fistula (n = 1).

Near-complete to complete recovery of nerve injury was reported for most patients, over a time period ranging from 4 months to 2 years [20, 23, 27, 28]. Thermal protection measures, such as hydrodissection or gas dissection, as well as neuromonitoring with electrostimulation were employed in most of the studies where the risk of nerve injury was deemed to be high [20, 23, 27, 28].

Several studies considered neural structures <1 cm from the tumour margin to be in close proximity [25, 27, 28]. Kurtz et al. suggested using CT or MRI, with complementary ultrasound guidance, for at risk procedures [24]. Saltiel et al. recommended a combined ultrasound-guided open surgery approach instead of percutaneous approach for at risk patients [25]. Shmitz et al. also employed staged cryoablation to minimise the risk of neural injury [22].

The most common minor complications included pain, swelling, haematoma, frostbite and sensory deficit.

### Efficacy

#### Tumor size & progression free survival

Tumor response was assessed by mRECIST in 4 studies [20, 24, 27, 28], RECIST 1.1 in 3 studies [21–23], MRI contrast product uptake in 1 study [24], and total lesional volume (TLV) and/or viable tumor volume (VTV) in 8 studies [20–23, 25–28]. TLV is defined as the total volume of the tumor assessed by an ellipsoid formula, manual segmentation or automated software measurement. VTV is defined as the enhancing portions of the tumor measured in the same manner.

**Fig 4:**
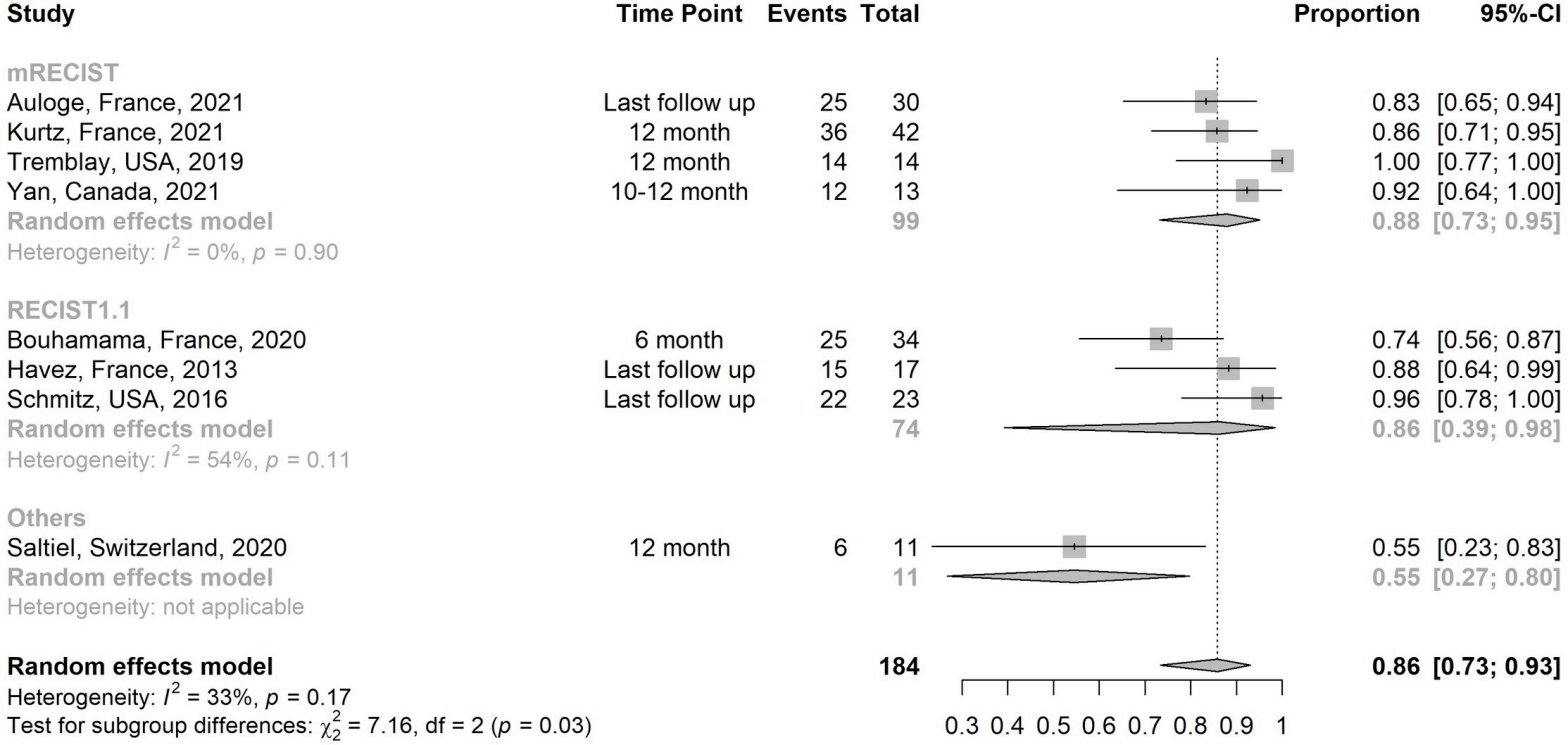
Subgroup analysis of the non-progressive disease rates of all studies.

The estimated pooled proportion of non-progressive disease rate of all studies is 85.8% (95% CI, 73.4–93.0; I^2^ = 32.9%) (Fig 4) [20–25, 27, 28].

**Fig 5:**
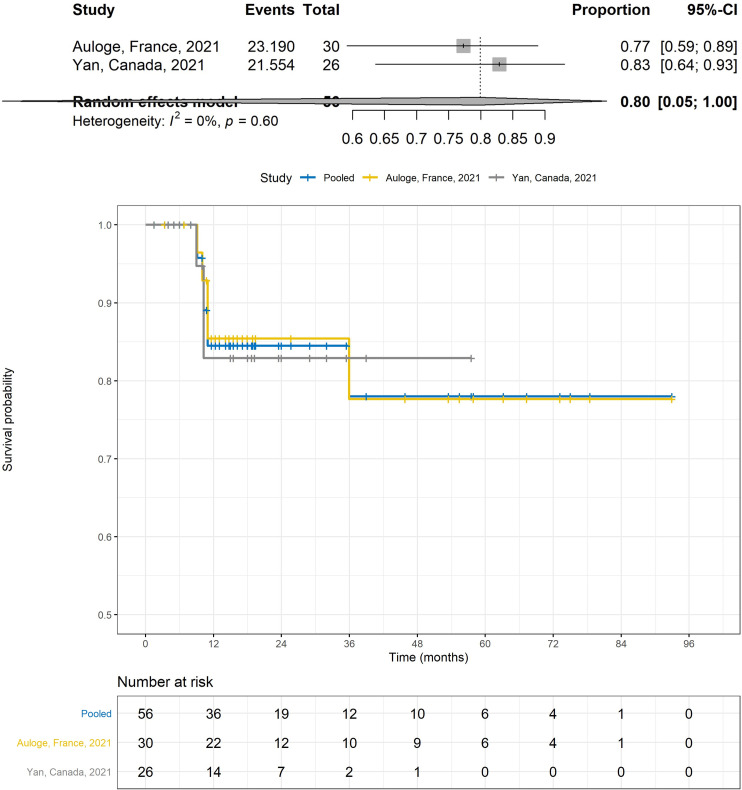
Pooled (Fig 5A) and individual survival curves (Fig 5B) using mRECIST for tumor response.

Data was too heterogeneous to combine and analyze overall post procedure TLV and VTV and are listed in Table 2. For patients treated with both curative and palliative intent, reported change in TLV and VTV at 6 months range from -6.8 to -73.5% [21, 23, 25, 28] and -44.2 to -65.6% respectively [21, 23, 25, 28]. Reported change in TLV and VTV at 12 months range from -56 to -66.6% [20, 25, 27] and 80% respectively [20, 25, 27]. For the combined PFS using mRECIST as an end point, median PFS was not reached for tumor progression at a median follow up of 16.7 months. The estimated PFS rate at 1 year was 84.5% (95% CI:74.6–95.8) and at 3 years 78.0% (95% CI: 63.8–95.3) (Fig 5A and 5B) [27, 28].

Kurtz et al. assessed PFS with mRECIST and symptom recurrence and reported that the median PFS was not reached with a median follow-up of 31 months after cryoablation [24].

Bouhamama et al. and Havez et al. had PFS curves determined by both symptom recurrence and tumor recurrence defined by RECIST 1.1 [21, 23]. Bouhamama et al. had disease free survival at 3 years of 42.2% [23]. Havez et al. had PFS at 1 and 2 years of 82.3% [21]. Saltiel et al. analysed PFS using viable tumor volume and reported PFS at 1 year of 62% [25]. No study reported 5-year survival. Factors identified on analysis by Kurtz et al. as statistically significant predictors of poor survival was tumor largest diameter [24].

For patients treated for curative intent, Auloge et al. reported CR (n = 13), PR (n = 3), SD (n = 1) and with PD (n = 2) [27]. Saltiel et al. reported CR (n = 6) and PR (n = 2) at 3 months, CR (n = 3) and, PR (n = 4), and PD (n = 1) at 6 months; and CR (n = 3), PR (n = 1), and PD (n = 4) at 12 months; change in TLV of -16.3% and -28.8% at 6 and 12months follow up respectively and change in VTV of -44.1% and +103.4% at 6 and 12months follow up respectively [25]. Yan et al. reported in 2 patients who achieved complete ablation, 1 patient had tumor recurrence at 6 months post procedure and 1 patient remained disease free at completion of the study (5 months) while median change in TLV and VTV based on the last follow up was -2.3% and -97.1% respectively [28]. Bouhamama et al. reported 9 patients had local recurrence and 14 patients had complete response [23]. Schmitz et al. reported 4.3% of tumors had tumor progression and 39.1% of tumors treated showed complete response [22]. Tremblay et al. reported 5 of 12 patients had complete ablation confirmed and showed sustained complete response at 12 months [20].

Some of the studies reported repeat ablations during the follow-up period as part of scheduled two-steps cryoablation, for residual disease, residual symptoms, recurrent disease and/or recurrent symptoms [21, 24, 25, 27, 28]. For recurrent and/or residual disease, Havez et al. reported 2 recurrences at 6 months, one of which was treated with repeat cryoablation [21]. Saltiel et al. reported 4 out of 14 procedures involved treatment of a residual lesion discovered at the 3-month follow-up [25]. Auloge et al. reported that 4 patients underwent a 2nd ablation procedure for the treatment of residual and/or recurrent disease, either to achieve complete ablation or for better local disease control, with the median time between the 1st and 2nd procedure being 14 months [27].

Yan et al. reported 10 of 25 patients (40%) underwent repeat cryoablation (18 of 44 procedures) for a mean of 10.6 months (range: 2–44.5 months) for relief of recurrent symptoms (3/18), as part of a 2 step procedure (2/18), treatment of residual symptoms and/or residual unablated tumor (9/18), local tumor progression (3/18) and for both symptom relief and local tumor progression (1/18) [28].

Kurtz et al. reported 5 patients had a 2-step ablation [24]. Schmitz et al. reported two patients had extra-abdominal desmoid tumors that received multiple treatments. Staged treatments of large extra-abdominal desmoid tumors were done for tumours located near critical structures [22].

There was no information on tumor size for the two step ablation.

### Symptom relief

#### Symptoms were generally defined as pain and/or functional impairment or disability by all studies

Functional status was assessed by Efrima et al. via a 36-Item Short Form Health Survey (SF-36) pre-operatively and at 12 months post-operatively for assessment of patient’s functional status [26]. Kurtz et al. analysed quality of life through a calculated utility score using the EQ5D-3L which consists of five dimensions and a visual analogic scale [24]. Bouhamama et al. utilised the Eastern Cooperative Oncology Group (ECOG) performance status [23].

2 studies reported pain improvement objectively using a visual analog scale (VAS) [23, 27].

9 studies with a total of 191 patients (89.3%, 191/214) had symptoms before cryoablation. Following cryoablation, a range of 37.5% to 96.9% of patients were reported to have experienced partial or complete symptom relief [20–28].

**Fig 6:**
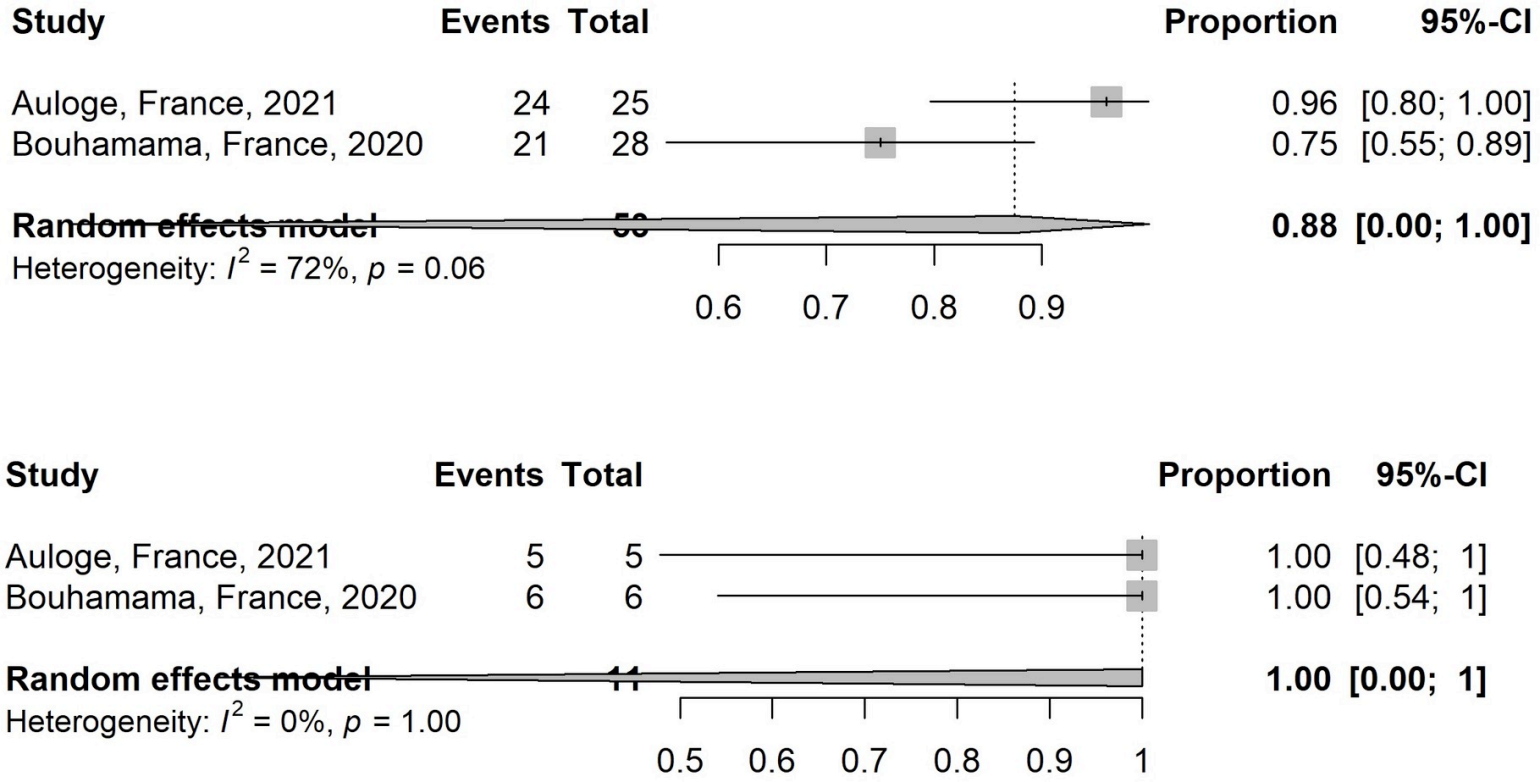
Comparison of the proportion of patients showing decrease in visual analogue scale (VAS) > = 3 for those with VAS > = 3 before treatment (Fig 6A) and proportion of patients showing total disappearance of pain for those with VAS <3 before treatment (Fig 6B).

2 studies assessed pain using a visual analog scale (VAS) and show a significant reduction in VAS scores post-treatment [23, 27]. These studies were analysed and the estimated pooled proportion of patients with decrease in VAS > = 3 for those with VAS > = 3 before treatment for these 2 studies is 87.5% (95% CI, 0.06–100; I^2^ = 71.5%) (Fig 6A). The estimated pooled proportion of patients with total disappearance of pain for those with VAS <3 before treatment is 100% (95% CI, 0–100; I^2 =^ 0%) (Fig 6B).

1 study assessed pain, quality of life and function by brief pain inventory score, VAS, monitoring frequency of analgesics intake and EQ5D-3L [24]. Two studies showed no significant difference in pain scores between the groups treated with complete and partial ablation [23, 24]. Five studies assessed pain using subjective inputs by patient [20–22, 25]. One study assessed symptoms using 36-Item Short Form Health Survey [26].

Symptom recurrence ranged from 18% to 21.2% [21, 28]. Yan et al. reported median time to symptomatic recurrence was 8 months (range: 3 to 24) and median time to symptom improvement was 2.5 months (range: 0.5 to 13) [28].

### Follow-up

Median follow-up ranged from 7 to 31 months (absolute range, 6 months to 7 years and 9 months). Mean follow up ranged from 11.3 to 53.7 months. Most studies conducted early postprocedural MRI within a month to establish a baseline for subsequent follow-up and detect partial ablation.

## Discussion

This review demonstrates that cryoablation is a suitable palliative treatment option because it has a low complication rate and provides durable short to medium term tumor response and symptom relief. In addition, as median PFS for tumor progression was not reached for the applicable studies in a range of median follow up ranging from 15.3 to 31 months [24, 27, 28], procurement of durable long term disease control may be achieved.

The estimated pooled proportion of non-progressive disease rate of 85.8% is comparable to current front line or alternative therapies, although the lack of long term data and heterogeneity of response criteria used by the selected studies in the review must be taken into consideration. Additionally, varying methods were used to assess tumor volume by the different studies, which could affect accuracy in tumor volume computation. However as these method were consistent within each study, these differences are unlikely to significantly affect the tumor response calculations.

Radiotherapy provides adequate long term disease control of 70% to 93% [36]. Chemotherapy achieves long term disease control of up to 50 to 70% depending on the series [12]. Other preferred therapies include imatinib, sorafenib (ORR 33%), pazopanib (83.7% of non-progression at 6 months) [37], and more recently gamma-secretase inhibitors (ORR 29%) [38]. However, these patients may suffer from protracted pain and disability. For example, the reported rates of late toxicity associated with radiotherapy range from 17 to 40%, with common late toxicities being skin and joint-related [9]. For chemotherapy, grade 3–4 toxicity rates of 17 to 93% have been reported [12], including peripheral neurotoxicity, hepatic toxicity, oral mucositis, and interstitial lung disease [39]. In a study comparing sorafenib with a placebo, grade 4 events that were associated with sorafenib included thrombocytopenia (2%) and anemia (2%) [2].

In patients treated with curative intent, a high rate of complete response (68.4%) was seen by Auloge et al. [27] as compared to other studies [20, 22, 23, 25, 28], although the median follow up was 18.5 months with a lack of long term data. Most of these studies showing complete responses did not report any subsequent local recurrences. MRI guidance was used by Auloge et al. as MRI defined the tumor margins better than CT and enabled greater accuracy in cases of curative intent [27]. However, some patients may require CT guidance in cases of large body habitus or very large tumor volume. The curative role of cryoablation when complete ablation can be achieved remains promising and more long term data is required to further evaluate its efficacy.

Despite the lack of standardization in reporting symptom relief, this review has shown that cryoablation can decrease symptoms and improve quality of life. Cryoablation can be performed for iterative symptomatic palliation in a setting of symptomatic recurrence.

Though this review has not established the safety of a combination therapy of cryoablation and chemoradiation in desmoid tumors, such combination therapies have been shown to be safe and well tolerated in other bone and soft tissue tumors and can be investigated in future studies [40–42].

The more recent studies have evaluated tumor response based on mRECIST rather than RECIST 1.1, as RECIST was deemed to not adequately identify all clinically relevant responses, and contrast enhanced MRI is the recommended modality for monitoring DT [12]. Volumetric signal and image texture assessment may allow early prediction of DT behaviour and tumor response [43].

A key advantage of cryoablation, over other ablation techniques, is that the iceball can be monitored with imaging, resulting in low complication rates. Not all extra-abdominal sites are technically feasible for cryoablation, with a craniofacial location deemed excessively high risk for neurovascular and cutaneous complications. Common complications include common peroneal nerve injury and skin necrosis. Operators should be careful when attempting cryoablation in the popliteal fossa. Apart from proximity of the DT to the skin, large tumor volumes and pre-cryoablation embolization were deemed to be risk factors for skin necrosis [27]. Techniques that have been employed in this series to mitigate risks include carbo or hydrodissection and neuromonitoring.

Limitations include the mostly retrospective nature of the selected studies (8/9), with heterogeneous patient selection, follow-up imaging protocols and varying assessments of tumor response and efficacy. Although there is a lack of high-quality data on cryoablation in extra-abdominal DT, current literature on cryoablation for DT when interpreted with caution does provide useful information. Long term efficacy of cryoablation cannot be confirmed on the basis of available data, as studies are heterogeneous in patient selection, methodology, and reporting. No study reported 5-year survival.

## Conclusion

In conclusion, percutaneous cryoablation is shown to be a safe and effective treatment for extra-abdominal DT with efficacy similar to those treated with traditional strategies in the short to medium term.

## Supporting information

S1 Checklist Preferred reporting items for systematic reviews and meta-analyses (PRISMA) checklist for conducting a systematic review. (DOCX)

S1 Table Inclusion and exclusion criteria reported by each included paper. (DOCX)

S2 Table Demographics and outcomes following cryoablation for desmoid tumors. (DOCX)

S1 File Appendix A: Modified Downs and Black checklist for the assessment of the methodological quality of both randomized and non-randomized studies. Appendix B: Funnel plots for assessment of risk of bias.
(DOCX)

## Abbreviations

DT: Desmoid tumor
FAP: Familial adenomatous polyposis
PRISMA: Preferred Reporting Items for Systematic Reviews and Meta-Analyses
VAS: Visual analogue scale
NSAIDs: Non-steroidal anti-inflammatory drug
TLV: Total lesional volume
VTV: Viable tumor volume
PFS: Progression-free survival
