# Supplementary material for: Cryotherapy in extra-abdominal desmoid tumors: A systematic review and meta-analysis
Source: PLoS One. 2021 Dec 23;16(12):e0261657. doi: 10.1371/journal.pone.0261657 (PMC8699690; doi:10.1371/journal.pone.0261657)
Supplement: S1 Table — (DOCX) [file pone.0261657.s002.docx]

Table 1 Inclusion and exclusion criteria reported by each included paper

| Study, Country of Origin, Year | Inclusion criteria | Exclusion criteria |
| --- | --- | --- |
| Yan, Canada, 2021 | - Biopsy proven progressive or symptomatic extra-abdominal DT and at least one cryoablation treatment between 25 February 2010 and 25 February 2020, with data available for follow up through February 2020.  - Pediatric patients defined as less than 18 years old.  - Patients with more than one treated tumor or who were treated in the same location more than once were included as a separate encounter | N.R. |
| Efrima, Israel, 2021 | - Patient over 18 years of age that had been treated with percutaneous cryosurgery by means of a three-phase protocol.  - Extra-abdominal desmoid tumors that showed progression of size in at least two sequential MRI scans executed 3 months apart.  - Patients with symptomatic desmoid tumors. | N.R. |
| Auloge, France, 2021 | - All patients with extra abdominal DT who underwent cryoablation from Jan 2008 to Nov 2019 were identified by research performed in our institutional Radiological Information System (Xplore, EDL, la Seyne-sur-Mer, France) with “Cryoablation” and “Desmoid tumor” entered together. | - Patients involved in a prospective study |
| Kurtz, France, 2021 | - Pathologically confirmed extra-abdominal DT.  - Progressive disease after at least two lines of adequate medical therapy [including tamoxifen, NSAIDs or chemotherapy], with functional symptoms and/or pain.  - Unresectable tumor or tumour amenable only to mutilating surgery deemed inappropriate in a NETSARC tumor board.  - Patients with mRECIST 1.1 criteria stable disease, but with persistent functional disability or tumor-induced pain not controlled by adequate pain medication including narcotics patients.  - Other criteria included: age ≥ 18 years old; tumor deemed accessible for cryoablation procedure by the radiologist operator of the investigating center (with 90% of destruction of the tumor achievable in one procedure of cryoablation and with a possible second cryoablation procedure if necessary and if scheduled at study entry); measurable lesion (mRECIST 1.1) using MRI (gadolinium injection mandatory); ECOG performance status 0-2; adequate biological and hematological parameters; affiliation to a medical insurance scheme for health costs coverage, and signed written informed consent. | - Contraindication for the procedure as stated by the interventional radiologist in terms of tumor size, proximity to neural/vascular structures making the procedure involving unacceptable risk  - Impaired hemostasis  - Concomitant participation in other experimental studies that could affect end-points of this study  - Contraindication to any form of sedation, MRI or gadolinium injection [proven allergy, glomerular filtration rate <30 ml/min by Modification of Diet in Renal Disease formula]  - Psychiatric disorders and adults under guardianship, pregnancy or breastfeeding, or under judicial protection. |
| Saltiel, Switzerland, 2020 | - Patients with histologically confirmed extra-abdominal DT treated with cryoablation and followed up with MRI before and after treatment. | - Intra-abdominal DT or no available follow-up. |
| Bouhamama, France, 2020 | - Extra-abdominal DT that was histologically proven by a percutaneous or a surgical biopsy.  - Surgery was contraindicated because it was considered too much mutilating. - Tumors were clinically or radiologically progressive despite of a systemic treatment (tamoxifen, pazopanib or non-steroidal anti-inflammatory drugs).  - Tumors were considered by a senior interventional radiologist as accessible to a percutaneous cryoablation on the preoperative imaging: visible with ultrasound or CT scan during procedure.  - Patients were included regardless of the size of the tumor. | N.R. |
| Tremblay, USA, 2019 | - Biopsy proven extra-abdominal DT who were treated with percutaneous cryoablation from July 2014 to May 2018, with follow up through January 2019. | N.R. |
| Schmitz, USA, 2016 | - Patients with extra-abdominal DT who underwent percutaneous cryoablation between June 15, 2004, and June 15, 2014. | N.R. |
| Havez, France, 2013 | - Presence of a symptomatic histologically-proven extra-abdominal DT  - Percutaneous cryoablation technically possible and en-bloc resection not possible (due to lesion location or size) or refused by the patient  - Cryoablation treatment option approved in a multidisciplinary team meeting. | N.R. |

Abbreviations: DT: desmoid tumor: NSAIDs: non-steroidal anti-inflammatory drugs; ECOG: Eastern Cooperative Oncology Group; N.R.: not reported
