# Supplementary material for: Cryotherapy in extra-abdominal desmoid tumors: A systematic review and meta-analysis
Source: PLoS One. 2021 Dec 23;16(12):e0261657. doi: 10.1371/journal.pone.0261657 (PMC8699690; doi:10.1371/journal.pone.0261657)
Supplement: S2 Table — (DOCX) [file pone.0261657.s003.docx]

Table 2 Demographics and Outcomes following Cryoablation for Desmoid Tumors

| Study, Country of Origin, Year | No. of Patients/Tumors/Procedures | Mean age (year) | Sex (M/F) | Curative/  Palliative Intent (patients) | Median Follow-up | Technical Success (%) | Major Complication (%) | Median Change in Tumor Volume | Progression Free Survival | Non-progressive disease rate | Symptom relief |
| --- | --- | --- | --- | --- | --- | --- | --- | --- | --- | --- | --- |
| Yan, Canada, 2021 | 25/26/44 | 32 | 8/17 | 10/15 | Imaging and clinical follow up is 6M and 10M respectively;  Follow up for DFS tumor recurrence and symptom recurrence is 15.3 and 21.0M respectively. | 100 | 2.4 | 4-6M:  TLV: -6.8%(NS)  VTV: -44.2% (p<0.05)  7-12M:  TLV: -6.7% (NS); VTV: -43.7% (p<0.05) | 3Y: 82.9% (tumor progression); 78.4% (symptom recurrence); Median DFS for tumor progression and symptom recurrence were not reached. | 10-12M: 92.3% | 96.9% |
| Efrima, Israel, 2021 | 11/11/16 | 35.3 | 5/6 | N.R. | 6M | N.R. | 0 | At last follow up:  TLV: -27.9%  VTV: -64.7% | N.R. | N.R. | 81% |
| Auloge, France, 2021 | 30/30/34 | 39^b^ | 9/21 | 19/11 | 18.5M | N.R. | 13.3 | At last follow up:  VTV: − 80%  1Y:  TLV: -66.6%  3Y:  TLV: −76.4% | 1Y: 85.1%  3Y: 77.3% | At last follow up: 83.3% | 96.7% |
| Kurtz, France, 2021 | 50/50/55 | 41 | 11/39 | N.R.; all patients aimed to have at least 90% tumor destruction | 31M (DFS) | N.R. | 15 of 50 patients had grade 3 or 4 side effects. No grade 5 side effects | N.R. | Median DFS not reached at median follow up | 12M: 86% | Significantly improved functional status and pain scores (BPI and EQ5D-3L) |
| Saltiel, Switzerland, 2020 | 10/10/14 | 33 | 1/9 | 8/2 | 53.7M^a^ | N.R. | 14.2 | 6M:  Curative intent: TLV: -16.3% VTV: -44.1%^a^  Palliative intent: TLV: +113.2% VTV: +148.6%^a^  12M:  Curative intent TLV: -28.8% VTV: +103.4%^a^  Palliative intent: TLV: +112.9% VTV: +192.1%^a^ | 3M: 90%  6M & 12M: 62% | 12M: 54.5% | 37.5% |
| Bouhamama, France, 2020 | 34/41/41 | 38 | 9/25 | 12/22 | 25M^a^ | N.R. | 4.8 | 6M:  TLV: -12.4% VTV: -65.6% (p<0.05)^a^ | 3Y: 42.2% | 6M: 73.5% | Decreased VAS score at 6 months |
| Tremblay, USA, 2019 | 23/23/30 | 40.5^b^ | 9/14 | 12/11 | 16.8M^a^ | 100 | 6.7 | 12M:  TLV: -56%  VTV: -80% ^a^ | N.R. | 12M: 100% | 90% |
| Schmitz, USA, 2016 | 18/26/31 | 39.9 | 8/10 | 18/0 | 16.2M^a^ | 100 | 0 | At last follow up:  TLV: -89.2% | N.R. | At last follow up: 95.7% | 80% |
| Havez, France, 2013 | 13/17/17 | 39.3 | 4/9 | 9/8^c^ | 7M | 100 | 5.8 | 3M:  TLV: -56%,  6M:  TLV: -73.5%,  At last follow up:  TLV: -87% | 6M, 12M & 24M: 82.3% | At last follow up: 88.2% | 76.9% |

Abbreviations: M: months; Y: years; NS: not significant; N.R.: not reported; DFS: disease free survival; BPI: Brief Pain Inventory; TLV: total lesion volume; defined by all the authors as volume of the tumor measured by ellipsoid formula, manual segmentation or software assisted, VTV: viable tumor volume; defined by all the authors as volume of the enhancing portions of the tumor measured by ellipsoid formula, manual segmentation or software assisted

^a^ Mean value, ^b^ Median value, ^c^ as per procedure.
