# Supplementary material for: Cryotherapy in extra-abdominal desmoid tumors: A systematic review and meta-analysis
Source: PLoS One. 2021 Dec 23;16(12):e0261657. doi: 10.1371/journal.pone.0261657 (PMC8699690; doi:10.1371/journal.pone.0261657)
Supplement: S1 File — Appendix A: Modified Downs and Black checklist for the assessment of the methodological quality of both randomized and non-randomized studies. Appendix B: Funnel plots for assessment of risk of bias. (DOCX) [file pone.0261657.s004.docx]

**Appendix A**

Modified Downs and Black checklist for the assessment of the methodological quality of both randomized and non-randomized studies[35]

| **Item** | **Criteria** | **Possible Answers** | |
| --- | --- | --- | --- |
| Reporting | | | |
| 1 | *Is the hypothesis/aim/objective of the study clearly described?* | Yes = 1 No = 0 | |
| 2 | *Are the main outcomes to be measured clearly described in the Introduction or Methods section?* | Yes = 1 No = 0 | |
| 3 | *Are the characteristics of the patients included in the study clearly described?* | Yes = 1 No = 0 | |
| 4 | *Are the interventions of interest clearly described?* | Yes = 1 No = 0 | |
| 5 | *Are the main findings of the study clearly described?* Simple outcome data (including denominators and numerators) should be reported for all major findings so that the reader can check the major analyses and conclusions. | Yes = 1 No = 0 | |
| 6 | *Have all important adverse events that may be a consequence of the intervention been reported (ie. peri-procedural complications)?* | Yes = 1 No = 0 | |
| 7 | *Have the characteristics of patients lost to follow-up been described?* | Yes = 1 No = 0 | |
| External validity | | |  |
| 8 | *Were the subjects asked to participate in the study representative of the entire population from which they were recruited?*. | Yes = 1  No = 0  Unable to determine = 0 | |

| 9 | *Were those subjects who were prepared to participate representative of the entire population from which they were recruited?* | Yes = 1  No = 0  Unable to determine = 0 | |
| --- | --- | --- | --- |
| 10 | *Were the staff, places, and facilities where the patients were treated, representative of the treatment the majority of patients receive?* | Yes = 1  No = 0  Unable to determine = 0 | |
| Internal validity - bias | | |  |
| 11 | *Was an attempt made to blind those measuring the main outcomes of the intervention?* | Yes = 1  No = 0  Unable to determine = 0 | |
| 12 | *If any of the results of the study were based on “data dredging”, was this made clear?* | Yes = 1  No = 0  Unable to determine = 0 | |
| 13 | *Were the statistical tests used to assess the main outcomes appropriate?* | Yes = 1  No = 0  Unable to determine = 0 | |
| 14 | *Were the main outcome measures used accurate (valid and reliable)?* | Yes = 1  No = 0  Unable to determine = 0 | |

**Appendix B**

Fig. 1 Funnel plot for pre-ablation tumor volume

Fig. 2 Funnel plot for pre-ablation tumor diameter

Fig. 3 Funnel plot for major complications

Fig. 4 Funnel plot for minor complications

Fig. 5 Funnel plot for tumor response
